# Attitudes and perceptions of dental students following the implementation of an online video and podcast compared to a traditional educational strategy in the endodontics course. A cross-sectional study

**DOI:** 10.21142/2523-2754-1401-2026-273

**Published:** 2025-12-28

**Authors:** Carmen Rosa García-Rupaya, Doris Maura Chacón Gonzales, Pedro Johan Caballero-García

**Affiliations:** 1 Dental surgeon. Endodontics specialist. Master's degree in Stomatology. Faculty of Dentistry, Universidad Nacional Federico Villarreal. Lima, Peru. cgarcia@unfv.edu.pe Universidad Nacional Federico Villarreal Dental surgeon. Endodontics specialist. Master's degree in Stomatology Faculty of Dentistry Universidad Nacional Federico Villarreal Lima Peru cgarcia@unfv.edu.pe; 2 Dental surgeon. Ortodontics specialist. Master's degree in Stomatology. Faculty of Dentistry, Universidad Nacional Federico Villarreal National University. Lima, Peru. dchacon@unfv.edu.pe Universidad Nacional Federico Villarreal Dental surgeon. Ortodontics specialist. Master's degree in Stomatology Faculty of Dentistry Universidad Nacional Federico Villarreal National University Lima Peru; 3 Bachelor of Science in Communication. Universidad Tecnológica del Perú. Lima, Peru. jcgfotografia@outlook.es Universidad Tecnológica del Perú Bachelor of Science in Communication Universidad Tecnológica del Perú Lima Peru jcgfotografia@outlook.es

**Keywords:** endodontics, video, podcast, attitudes, perceptions, endodoncia, video, podcast, actitudes, percepciones

## Abstract

**Objective:**

To evaluate the attitudes and perceptions of dental students following the implementation of an online video and podcast compared to a traditional educational strategy in the Endodontics course.

**Materials and Methods:**

This descriptive cross-sectional study was conducted among students enrolled in the Endodontics course. A measurement instrument was developed and validated through expert judgment; it consisted of fifteen items assessing attitudes and ten questions regarding perceptions. A 4.30-minute video was created on the topic of chamber access in maxillary teeth, and a 15-minute podcast was produced on pulpal lesions. Gender-based comparisons were conducted using the Wilcoxon test. Additionally, Chi-square and Fisher's exact tests were applied for individual item associations with the gender covariate. A significance level of α < 0.05 was adopted.

**Results:**

A total of 21 male students (38%) and 34 female students (62%) participated, with a mean age of 22.80 years. The average attitude score was 67.33, with a maximum of 75 and a minimum of 55 points. A statistically significant difference was found between the scores of male and female students (p = 0.025). An association was observed between attitudes toward the use of the video (p = 0.049) and the podcast (p = 0.041) and the student’s gender. Additionally, 67.27% of students perceived that all three educational strategies should be used in the teaching process of endodontics. Students identified strengths in both the video and podcast, with no reported weaknesses.

**Conclusion:**

The use of video and podcast in endodontics may serve as a valuable alternative to complement the content provided in undergraduate dental education.

## RESUMEN

**Objetivo:**

Evaluar las actitudes y percepciones de estudiantes de odontología tras la aplicación de un video y un *podcast* en línea frente a una estrategia educativa tradicional en el curso de endodoncia.

**Materiales y métodos:**

: Este estudio descriptivo transversal se realizó en la población de alumnos del curso de endodoncia. Se construyo y validó mediante juicio de expertos un instrumento de medición, el cual constó de quince ítems de actitudes y diez preguntas de percepciones. Se elaboró un video sobre el tema de acceso cameral en piezas dentarias superiores, con una duración de 4:30 minutos, y se creó un *podcast* sobre lesiones pulpares, con una duración de 15 minutos. Para las comparaciones según género, se utilizó la prueba de Wilcoxon. Adicionalmente, se emplearon el chi cuadrado y la prueba exacta de Fisher para las asociaciones de género. Se trabajó con un nivel de significancia de α < 0,05.

**Resultados:**

Se incluyó a 21 hombres (38%) y 34 mujeres (62%), con una edad promedio de 22,8 años. La media del puntaje de actitudes fue de 67,33, siendo el mayor de 75 puntos y el menor de 55 puntos. Se encontró diferencias significativas entre el puntaje de hombres y mujeres, con un p = 0,0250. Se observó una asociación entre las actitudes hacia el uso del video (p = 0,049) y el *podcast* (p = 0,041) y el género del estudiante. Un 67,27% de los estudiantes percibieron que deben utilizarse las tres estrategias educativas en el proceso de enseñanza en endodoncia. Los estudiantes percibieron fortalezas en cuanto al video y el *podcast*, sin presencia de debilidades.

**Conclusión:**

: El uso del video y el *podcast* en endodoncia puede ser una buena alternativa para complementar los contenidos brindados para la formación del estudiante de pregrado.

## INTRODUCTION

During the pandemic, online education was implemented and proved to meet a large portion of students’ educational needs. Even prior to this event, for more than a decade, information strategies such as the use of videos, podcasts, and others had already been incorporated ^1^^-^^3^. Nowadays, university students frequently use various technologies to manage information, requiring educators to update their teaching strategies so that course content is delivered in an engaging and innovative manner ^4^^,^^5^.

Numerous studies have demonstrated the superiority of “video-assisted clinical instruction” over traditional teaching, particularly for the development of clinical competencies ^1^^,^^4^^-^^6^.

Dental education has undergone a transformation with the goal of enhancing students’ problem-solving skills. In this regard, information and communication technologies (ICTs) play a key role through the incorporation of online platforms as alternative teaching methods ^7^.

For this recent generation, educational videos must be produced with attention to both aesthetics and functionality in order to capture students’ attention, making video clips an effective learning tool ^1^^,^^6^.

The emergence of COVID-19 brought the challenge of teaching practical skills via online learning. The experience of the University of Applied Sciences in Bern emphasized the use of highly enhanced course content delivered through technological platforms ^5^. Although dental training cannot rely solely on distance learning, certain complementary strategies can be integrated with traditional methods to enhance learning outcomes ^3^.

Alternative methods were explored to deliver curriculum content. Among them, classes were adapted and distributed via video platforms such as YouTube. Likewise, podcasts became valuable tools for remote learning and have since gained increasing popularity among students ^4^. In medical education, their advantages and practical applications have also been explored to narrow the gap between technological innovations and effective teaching ^8^. Given that podcasts can be accessed not only via computers but also smartphones, they are a highly versatile and accessible technology for both students and instructors ^9^.

Furthermore, students often experience idle periods between classes or during commutes, which could be used to listen to podcasts, helping to balance academic and personal life while reducing stress ^10^^,^^11^.

A systematic review including twelve studies reported that dental students had positive perceptions of innovative teaching strategies, especially those implemented in dental materials course, which led to improved learning outcomes compared to traditional lectures ^12^.

A study comparing three teaching methods in oral medicine, traditional, flipped classroom, and online, found that the online approach helped students acquire more knowledge and positively influenced their clinical practice ^13^.

Similarly, a cross-sectional study conducted in Southeast Asian countries showed that dental students acknowledged the benefits of online learning, including flexible scheduling and improved communication through various platforms, leading to more favorable perceptions of this modality as a complement to traditional instruction ^14^.

Researchers at a university in Iran's Department of Oral Medicine and Surgery conducted a mixed-methods study (qualitative and quantitative) to analyze the integration of videos in their courses. They found that while faculty viewed online education as an opportunity, they emphasized the need for adequate infrastructure and training for both instructors and students to fully utilize its benefits ^15^.

From the professional perspective, Eleman et al. ^16^ implemented an online course for dentists, observing high satisfaction levels and a strong willingness to recommend it, due to both content quality and technological advantage, highlighting the professors' academic caliber.

Prakash et al. ^17^ reported that podcasts offer several advantages in health sciences education. They classified podcasts by duration: short (1-5 minutes), medium (6-15 minutes), and long (>15 minutes). Students preferred those of medium length for reviewing content prior to evaluations.

In dental school courses, both theoretical and practical content are typically delivered using PowerPoint presentations and clinical demonstrations. Additional content is reinforced through current scientific articles, making it crucial that students have access to this information through various channels. This underscores the importance of producing videos specifically tailored to each course, as well as utilizing podcasts, versatile tools that students can access at any time and place.

For this reason, it is relevant to investigate students’ acceptance of these technologies, given a noticeable gap in the scientific literature. In this way, the aim of this study was to evaluate the attitudes and perceptions of dental students following the implementation of an online video and podcast compared to a traditional educational strategy in the Endodontics course.

## MATERIALS AND METHODS 

This cross-sectional study was conducted at the Faculty of Dentistry of the Universidad Nacional Federico Villarreal, Lima Peru in 2024. The sample (n = 55) was census-based and included seventh-semester students officially enrolled in the Endodontics course.

The research project was reviewed and approved by the Ethics Committee of the Faculty of Dentistry at Universidad Nacional Federico Villarreal under resolution code No. 004-2024-Ethics Committee-FO-UNFV, prior to the execution of the study. All students voluntarily agreed to participate by signing an informed consent form.

An instrument was developed to measure students' attitudes and perceptions regarding the use of online educational strategies. It was based on previously validated instruments used in the studies of Baris Sezer ^18^, Peart et al. ^19^, and Abdullatif Kaban ^20^. For the present research, the instrument was validated only through expert judgment by six faculty members with more than ten years of teaching experience in dentistry, including thesis advising and research project development, and holding specialist, master’s, or doctoral degrees. As a result of this content validation, the Aiken’s V coefficient was calculated, yielding a value of 0.98 for the attitudes questionnaire and 0.99 for the perceptions questionnaire. 

The educational strategies were specifically designed for this study and covered topics included in the official Endodontics syllabus (access cavity preparation and pulpal lesions), which are also covered in traditional lectures.

Video Production: The video addressed the topic of access cavity preparation in maxillary teeth and was recorded in the Endodontics Laboratory. The demonstration was performed by the principal investigator. It showed the location and process for creating access cavities in an incisor, a first premolar, and a first molar. The total duration of the video was 4 minutes and 30 seconds. Audio was standardized using an AI voice-over application.

Podcast Production: The podcast covered the topic of pulpal lesions and featured the principal investigator-an Endodontics specialist-as the host, and a guest faculty member with more than ten years of experience in Endodontics. A guide was prepared based on the 2009 American Association of Endodontists (AAE) classification of pulpal lesions, which is still in effect. The podcast discussed reversible pulpitis, irreversible pulpitis, pulpal necrosis, previously initiated tooth, and previously treated tooth. The final duration was 15 minutes.

Both the video and the podcast were recorded and edited by a professional audiovisual communicator. A Sony Full Frame camera was used for primary shots, and an iPhone 15 Pro Max was used for secondary shots focused on the interviewer and interviewee. Editing was performed using Final Cut Pro X on a Mac Mini M2 Pro computer, with 4K resolution. The audiovisual editor was responsible for video and audio production, music integration, color grading, and overall post-production of the video and podcast.

The educational video and podcast were projected live to the Endodontics students. Immediately afterward, a coordinated session was held for students to complete the questionnaires on attitudes and perceptions regarding the online strategies.

### Statistical analysis

A database was created in Excel and processed using STATA version 18. Attitudes were measured based on their intensity using a Likert scale, with scores ranging from 15 to 75 across 15 items. Perceptions were evaluated using ten individual items, with results expressed in relative frequency (%).

The statistical analysis for qualitative variables such as sex were presented using absolute and relative frequency distributions, while age was expressed as mean ± standard deviation. Inferential analysis of attitudes included quantitative assessment of each item’s scores, with results presented as means, standard deviations, and minimum/maximum values. Gender-based comparisons were conducted using the Wilcoxon test. Additionally, Chi-square and Fisher's exact tests were applied for individual item associations with the gender covariate. A significance level of α < 0.05 was adopted.

For perceptions, absolute and relative frequencies were calculated for each item.

## RESULTS

In this study, female participants were predominant, comprising 62% of the sample. The mean age was 22.80 years, with a minimum age of 20 and a maximum of 29 years.

The Likert scale was used to assess the intensity of students' attitudes toward the educational strategies. This scale consisted of 15 items, with scores ranging from a minimum of 55 points to a maximum of 75 points. The mean score was 67.32 (Table 1).

**Table 1:** Description of Attitude Scores of Endodontics Course Students (2024-1) Regarding the Use of Videos and Podcasts as Complementary Educational Strategies

Attitudes	n	Mean	SD	Min	Max
Item 1	55	4.73	0.45	4	5
Item 2	55	4.73	0.45	4	5
Item 3	55	4.33	0.67	2	5
Item 4	55	4.62	0.56	3	5
Item 5	55	4.76	0.43	4	5
Item 6	55	4.64	0.52	3	5
Item 7	55	4.60	0.56	3	5
Item 8	55	4.26	0.69	2	5
Item 9	55	4.55	0.57	3	5
Item 10	55	4.73	0.45	4	5
Item 11	55	4.40	0.56	3	5
Item 12	55	4.31	0.61	3	5
Item 13	55	4.09	0.75	2	5
Item 14	55	4.31	0.57	3	5
Item 15	55	4.36	0.56	3	5
Total Score	55	67.33	5.61	55	75

A statistically significant difference was observed between the scores obtained by male and female participants in the study sample (Table 2). An association was also identified between gender and responses to item 4 and item 10 (Table 3).

**Table 2:** Comparison of Attitude Scores of Endodontics Course Students (2024-1) Regarding the Use of Videos and Podcasts as Complementary Educational Strategies, by Gender

Gender	n	Promedio	DE	p
Male	21	65.33	6.43	
Female	34	64.68	5.82	0.0250

Wilcoxon test

**Table 3:** Attitudes of Endodontics Students (Semester 2024-1) Toward the Use of Videos and Podcasts as Complementary Educational Strategies, by Gender

ATTITUDES	Gender	Disagree / Neutral n (%)	Agree n (%)	Strongly Agree n (%)	p-value
1. The video information was clear	Male	--	8 (14.55)	13 (23.64)	0.157
	Female	--	7 (12.73)	27 (49.09)	
2. The video content was interesting	Male	--	7 (12.73)	14 (25.45)	0.428
	Female	--	8 (14.55)	26 (47.27)	
3. The video duration was appropriate	Male	2 (3.64)	13 (23.64)	6 (10.91)	0.306*
	Female	2 (3.64)	15 (27.27)	17 (30.91)	
4. I learned a lot from the video	Male	1 (1.82)	10 (18.18)	10 (18.18)	0.049*
	Female	1 (1.82)	7 (12.73)	26 (47.27)	
5. I would use this video to reinforce my knowledge	Male	--	7 (12.73)	14 (25.45)	0.183
	Female	--	6 (10.91)	28 (50.91)	
6. The podcast information was clear	Male	1 (1.82)	9 (16.36)	11 (20.00)	0.174*
	Female	--	9 (16.36)	25 (45.45)	
7. The podcast content was interesting	Male	1 (1.82)	10 (18.18)	10 (18.18)	0.099*
	Female	1 (1.82)	8 (14.55)	25 (45.45)	
8. The podcast duration was appropriate	Male	4 (7.27)	10 (18.18)	7 (12.73)	0.347*
	Female	2 (3.64)	18 (32.73)	14 (25.45)	
9. I learned a lot from the podcast	Male	1 (1.82)	11 (20.00)	9 (16.36)	0.155*
	Female	1 (1.82)	10 (18.18)	23 (41.82)	
10. I would use this podcast to reinforce my knowledge	Male	--	9 (16.36)	12 (21.82)	0.041
	Female	--	6 (10.91)	28 (50.91)	
11. The traditional class information was clear	Male	1 (1.82)	14 (25.45)	6 (10.91)	0.160*
	Female	1 (1.82)	15 (27.27)	18 (32.73)	
12. The traditional class content was interesting	Male	1 (1.82)	16 (29.09)	4 (7.27)	0.039*
	Female	3 (5.45)	14 (25.45)	17 (30.91)	
13. The traditional class duration was appropriate	Male	4 (7.27)	12 (21.82)	5 (9.09)	0.772
	Female	5 (9.09)	18 (32.73)	11 (20.00)	
14. I learned a lot from the traditional class	Male	1 (1.82)	16 (29.09)	4 (7.27)	0.077*
	Female	2 (3.64)	16 (29.09)	16 (29.09)	
15. I would revisit this traditional class to reinforce my knowledge	Male	--	14 (25.45)	7 (12.73)	0.414*
	Female	2 (3.64)	17 (30.91)	15 (27.27)	

*Fisher’s Exact Test *Chi-square test

According to Table 4, students perceived that the practical topic in the course was better addressed through the video format (47.27%), while the theoretical content was best delivered through the podcast (47.27%). Furthermore, 87.27% of students agreed that online didactic strategies met their expectations.

**Table 4:** Perceptions of Endodontics Students (Semester 2024-1) Regarding Content and Duration of Videos and Podcasts as Complementary Educational Strategies

Perception Questions	Podcast n(%)	Video n(%)	Traditional Class n(%)	All Three Equally n(%)
2. Was the content of the practical topic in Endodontics better with?	10 (18.18)	26 (47.27)	3 (5.45)	16 (29.09)
3. Was the content of the theoretical topic in Endodontics better with?	26 (47.27)	13 (23.64)	2 (3.64)	14 (25.45)
4. Is the duration time very important to maintain attention in?	11 (20.00)	14 (25.45)	2 (3.64)	28 (50.91)
5. Does the delivery of classes with online didactic strategies meet expectations?	4 (7.27)	3 (5.45)	--	48 (87.27)

As shown in Table 5, students agreed that the educational strategies (podcast, video, and traditional class) were effective in terms of content delivery, clarity, and the presentation of PowerPoint slides.

**Table 5:** Perceptions of Endodontics Students (Semester 2024-1) Regarding the Strengths of Using Videos and Podcasts as Complementary Educational Strategies

Perception Questions	Clarity n(%)	Content Management n (%)	PPT Presentation n (%)	All Aspects n (%)
6. What is your best perception of video implementation as an online didactic strategy?	9 (16.36)	13 (23.64)	5 (9.09)	28 (50.91)
7. What is your best perception of podcast implementation as an online didactic strategy?	4 (7.27)	19 (34.55)	4 (7.27)	28 (50.91)
8. What is your best perception of traditional class implementation as a didactic strategy?	7 (12.73)	13 (23.64)	9 (16.36)	26 (47.27)

Table 6 shows that students expressed mixed opinions about the duration of the educational strategies, with approximately half perceiving time as a weakness. However, no weaknesses were identified in the video format, with 81.82% of students indicating no issues.

**Table 6:** Perceptions of Endodontics Students (Semester 2024-1) Regarding Weaknesses in the Use of Videos and Podcasts as Complementary Educational Strategies

Perception Questions	Duration Time n(%)	Accessibility Difficulty n(%)	Unclear Audio n(%)	All Weaknesses n (%)	None n (%)
9. What is your perception of the weaknesses in podcast implementation as a didactic strategy?	25 (45.45)	4 (7.27)	1 (1.82)	--	25 (45.45)
10. What is your perception of the weaknesses in video implementation as a didactic strategy?	8 (14.55)	2 (3.64)	--	--	

## DISCUSSION

For this study, a video and a podcast were developed as complementary educational strategies to a traditional class in the Endodontics course. An instrument was constructed to assess students' attitudes and perceptions toward the use of these educational tools. Attitudes were measured using a Likert scale, while perceptions were gathered through closed-ended questions related to characteristics such as duration, content, audio quality, and others.

Podcasts are highly versatile, as they can combine visual and auditory elements or both, which increases student engagement with this technology ^21^. A study conducted in Peru on the contribution of podcasts to dental education reported that access to podcasts was widespread, as they could be played on various devices including laptops, tablets, and even mobile phones ^22^.

In the present study, the podcast focused on pulpal lesions and featured a mix of visual and auditory information in the format of a dialogue between two specialists. Supporting images were included to reinforce the discussion. The final duration was 15 minutes. This differs from the study by Saravia, which was conducted in the field of restorative dentistry and involved three audio-only podcasts of less than 15 minutes each, consisting of the narrated reading of PDF articles. Another study conducted in Ecuador produced two podcasts of 3 minutes and 53 seconds on endodontic topics, though it did not specify the characteristics of the materials used ^10^.

Unlike the studies mentioned above, the present research involved an audiovisual communication specialist as a co-investigator responsible for recording, editing, standardizing audio, and including visual reinforcements aligned with the content. This ensured a more dynamic presentation and professional-level production quality.

Regarding videos, they can serve as valuable complement to traditional lectures. Instructors may use publicly available videos or create original content aligned with course objectives. Dong proposes twelve key considerations for effective educational video creation, including minimizing cognitive overload, limiting duration to under 15 minutes, and maintaining high standards in both visual and audio quality ^23^.

In this study, the video topic was access cavity preparation in artificial maxillary teeth. The demonstration was performed by a certified Endodontics specialist and had a total duration of 4 minutes and 30 seconds. To ensure audio consistency, a voiceover was generated using artificial intelligence software. The editing process was led by an audiovisual specialist. This differs from a study conducted in Tehran, where videos were recorded directly on patients with and without expert commentary. These addressed topics such as cavity access, canal shaping, and root canal obturation, and students’ knowledge was assessed afterward ^(^¹^)^. In contrast, the present study measured attitudes and perceptions rather than learning outcomes.

Other researchers have opted to use videos already available on free platforms like YouTube, with one study reporting that such videos were well received by dental students in the area of Periodontics ^24^. However, many times these resources do not fully align with the specific course content being taught ^25^. This contrasts with the current study, where the video was specifically produced in accordance with the Endodontics course guidelines at the faculty.

In the present study, students generally responded with “agree” and “strongly agree” regarding the use of videos. This aligns with a study conducted in Korea, where students also indicated that they liked the videos and would be willing to rewatch them. However, it should be noted that the videos in that study were sourced from YouTube ^4^.

There is limited prior research evaluating student attitudes and perceptions toward self-produced videos in the Endodontics course, making it difficult to draw direct comparisons. This highlights the need for future research that compares original videos and podcasts with publicly available content.

In relation to podcasts, the current findings are consistent with those reported by Sacoto et al., who found highly positive student attitudes, mostly “strongly agree”, regarding podcast use in dental education^10^. This contrasts with the study by Weib, in which 58.8% of nutrition students indicated they would not use podcasts ^26^.

Regarding content quality and clarity in podcasts, Sacoto ^10^ reported very high percentages of “strongly agree” responses, up to 56.8%, which aligns with our findings. This may be attributed to the fact that both studies involved dental students and were conducted recently. Nevertheless, it would be valuable to explore further research on podcast duration and content selection.

In the present study, a quantitative assessment of attitudes was conducted by assigning scores to each item. The total mean score was 67.32, with a maximum score of 75, indicating that students were generally in favor of implementing these strategies in their learning process. According to the qualitative interpretation of the Likert scale, most responses leaned toward “strongly agree.” Statistically significant differences were observed between male and female students, though not between different age groups. Future studies should consider incorporating other relevant covariates.

Wei See et al. ^27^ noted that in modern educational environments, there is a noticeable shift toward blended learning, which combines traditional methods with various online strategies. Educational videos and podcasts are becoming viable tools that align with the digital literacy of new generations of students and instructors who are seeking to improve higher education practices. Therefore, strengthening such auxiliary tools is essential in the training of future dental professionals.

This research provides two main contributions: (1) the design and content validation of a specialized measurement instrument, and (2) the production of audiovisual content tailored to the official syllabus of the Endodontics course. The findings reflect positive attitudes and perceptions from students regarding the integration of these strategies into their own professional education. As such, these tools should be considered for implementation across other courses in the dental curriculum.

### Limitations

One limitation of the study is that there are no previous studies with videos and/or podcasts specifically developed for educational research, which makes comparisons difficult.

## CONCLUSION

It is concluded that students perceived these educational strategies to be well-edited, clear, and well-organized in terms of content. The high attitude scores suggest that video and podcast formats represent valuable alternatives to complement undergraduate endodontic education.

## Financing:

SSelf-financed
